# Successful use of olorofim for the treatment of *Lomentospora prolificans* knee tendonitis, synovitis, and concomitant osteomyelitis in an immunocompetent child

**DOI:** 10.1128/aac.00602-25

**Published:** 2025-08-27

**Authors:** Mary Fortini, Trahern W. Jones, Tristan R. Grams, Sonia Mehra

**Affiliations:** 1 Department of Pediatrics, Infectious Diseases Section, University of Utah and Primary Children’s Hospital 161528, Salt Lake City, Utah, USA; 2 Department of Pathology-Medical Microbiology, University of Utah 7060, Salt Lake City, Utah, USA; Houston Methodist Hospital and Weill Cornell Medical College, Houston, Texas, USA

**Keywords:** olorofim, *Lomentospora prolificans*, fungal osteomyelitis, *Scedosporium *infections, resistant fungal infections

## Abstract

*Lomentospora prolificans* is a filamentous mold that can cause infections in both immunocompromised and immunocompetent individuals. Infection can be either focal or disseminated, often acquired via inhalation or direct conidia inoculation (A. Konsoula, C. Tsioutis, I. Markaki, M. Papadakis, A.P. Agouridis, N. Spernovasilis. Microorganisms 10:1317, 2022, https://doi.org/10.3390/microorganisms10071317). This pathogen is challenging to treat due to its high MICs to available antifungals. We present a case of an 8-year-old previously healthy female who developed *L. prolificans* knee tendonitis and synovitis with patellar osteomyelitis after a flat patio stone laceration in Southern California, who ultimately was treated with the novel antifungal, olorofim.

## CASE PRESENTATION

**Fig 1:**
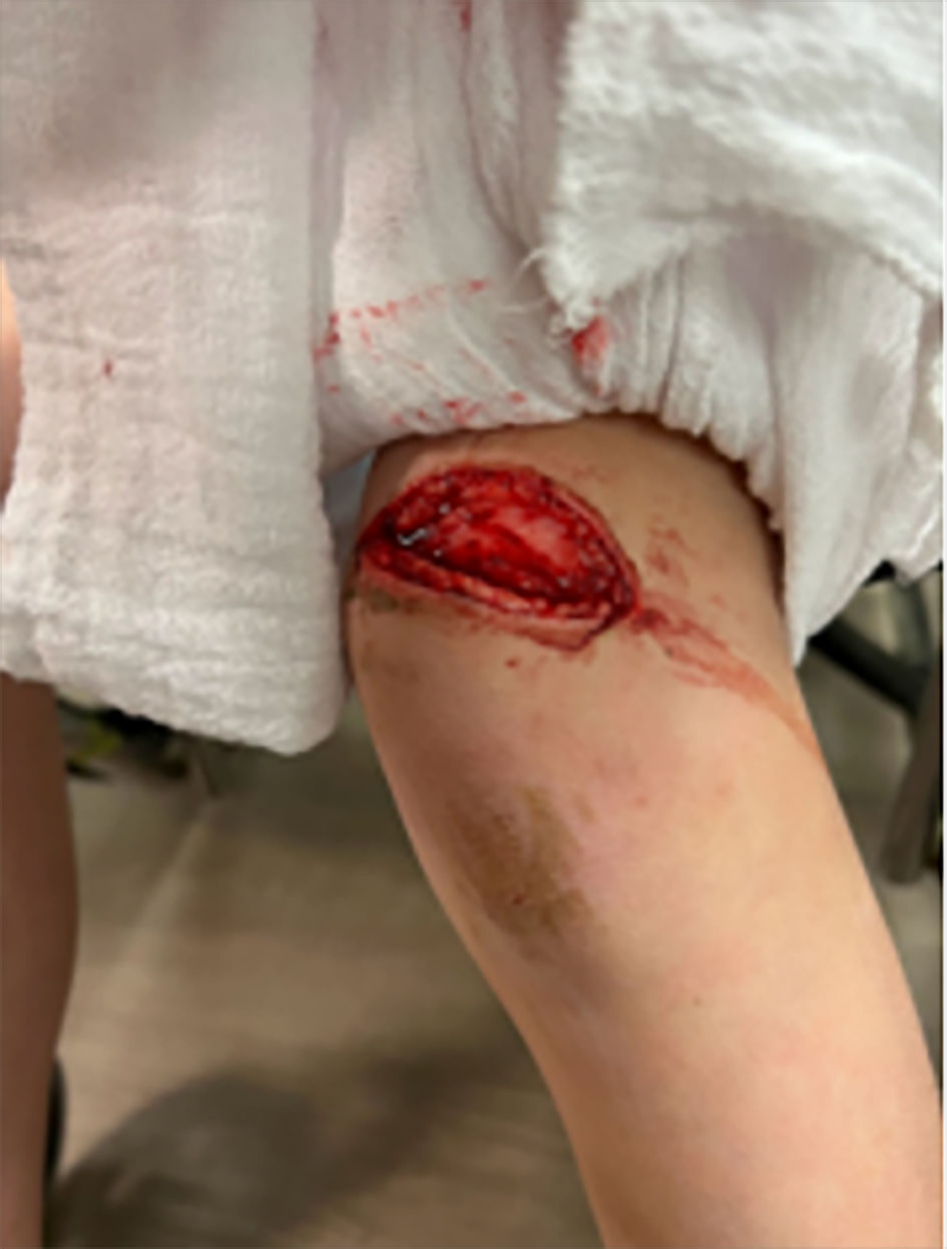
Initial injury, approximately 3–4 inches long and approximately 1 inch wide.

An 8-year-old previously healthy immunized female fell on a flat patio stone in Southern California, resulting in a left knee laceration with visible bone (Fig. 1). She was taken to a local Emergency Department (ED), where the wound was cleaned with iodine and sutured. An x-ray was performed, which was normal. She was sent home with cephalexin, but about 3 days into her antibiotic course, she developed fever, pain, swelling, and erythema on her left knee, prompting another visit to the ED. No imaging or cultures were done during this second visit, but the cephalexin was transitioned to clindamycin for a 10-day course. She was sent home with resolution of her erythema; however, she had minimal improvement in her swelling and pain. About a month later, she was brought to the ED again due to ongoing swelling and pain. An x-ray revealed a large left knee joint effusion with overlying soft tissue swelling. Complete blood count with differential at the time was unremarkable; however, her inflammatory markers were elevated with a C-reactive protein (CRP) of 11.5 mg/dL (reference range, 0–1 mg/dL) and an erythrocyte sedimentation rate (ESR) of 63 mm/h (reference range, 0–20 mm/h). MRI of the left knee with and without contrast revealed osteomyelitis of the patella, hyper-enhancing synovium, and significant inflammation of the thigh muscles and patellar tendon. Orthopedic Surgery performed a bedside arthrocentesis with fluid showing a white blood cell count of 9,523 cells/μL with neutrophilic predominance of 89% and red blood cells of 162,000 cells/μL. Gram stains of the fluid were negative; no fungal or acid-fast bacilli (AFB) cultures were obtained.

**Fig 2:**
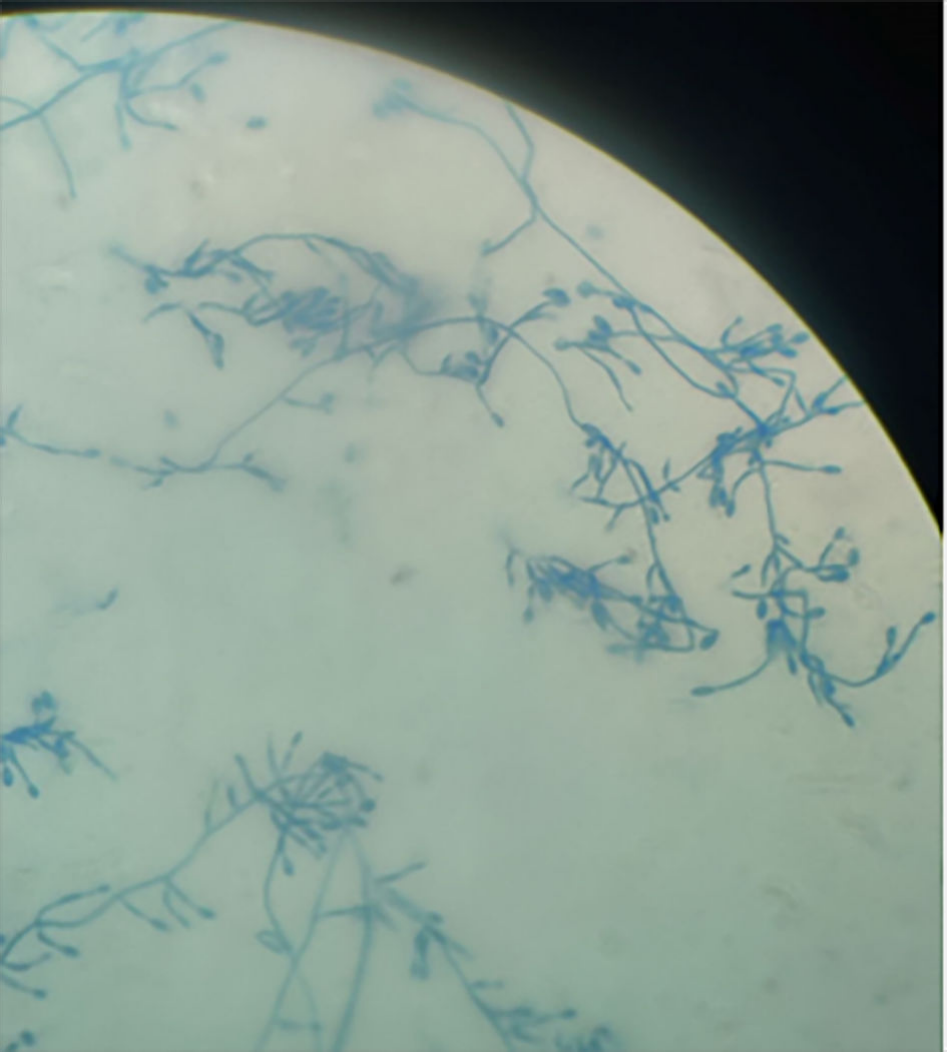
Adhesive tape preparation of *L. prolificans* stained with lactophenol cotton blue, visualized at 400× magnification. The hyphae are septate and pigmented, characteristic of dematiaceous fungi. Conidiogenous cells (annellides) display a swollen basal region with an elongated, tapering neck distinguishing it from *Scedosporium* species. Conidia are arranged in small clusters at the apices of the annellides and are unicellular, smooth-walled, and ovoid, possessing a slightly narrowed, truncate base.

The day following her arthrocentesis, the patient was subsequently taken by Orthopedic Surgery for an incision and drainage of her knee. No purulence was encountered during this procedure; however, there was significant inflammation noted in the synovial lining. Synovial fluid, synovial tissue, and patellar bone were sent for aerobic, anaerobic, AFB, and fungal cultures, and she was started on cefazolin, 50 mg/kg IV q8h. Her synovial fluid, synovial tissue, and patellar bone aerobic and fungal cultures ultimately all grew mold, identified as *Lomentospora prolificans* via matrix-assisted laser desorption ionization time-of-flight mass spectrometry (Fig. 2). Additionally, her prior aerobic culture from her arthrocentesis identified the same fungal organism about 3 days after her arthrocentesis was performed.

After cultures turned positive for mold, but before the specific organism was known, she was started on liposomal amphotericin B, 5 mg/kg IV q24h; however, after mold identification of *L. prolificans*, and upon literature review, this was transitioned to voriconazole, 9 mg/kg po BID for two doses, then 8 mg/kg po BID for maintenance dosing, and micafungin, 3 mg/kg IV q24h. Terbinafine, 125 mg po BID, was added a few days later while awaiting results of extended susceptibilities, performed at the University of Texas Health Science Center at San Antonio. As the patient had defervesced, she still had an elevated, although downtrending CRP; however, due to improving clinical status and labs, she was discharged home with close Infectious Diseases and Orthopedics outpatient follow-up.

Susceptibilities (performed via reference broth microdilution testing using a frozen broth microdilution panel with CLSI M38 used as a reference to set up testing of the isolates) returned following this patient’s discharge and showed the following: amphotericin B (MIC: 8 μg/mL), anidulafungin (MIC: 2 μg/mL), caspofungin (MIC: 4 μg/mL), fluconazole (no MIC reported, but noted to be resistant based on the CLSI M38M51S-ED3 guidance), isavuconazole (MIC: ≥16 μg/mL), itraconazole (MIC: ≥16 μg/mL), micafungin (MIC: 4 μg/mL), posaconazole (MIC: ≥16 μg/mL), voriconazole (MIC: ≥16 μg/mL), ibrexafungerp (MIC: >8 μg/mL), rezafungin (MIC: >8 μg/mL), terbinafine (MIC: >2 μg/mL), voriconazole + terbinafine synergy (MIC: >4 μg/mL + >2 μg/mL), and olorofim (MIC: 0.06 μg/mL).

## MULTIPLE CHOICE QUESTION

**Which antifungal would be the best choice to treat, given the previously noted MICs?**

**(A) An echinocandin**

**(B) Voriconazole + terbinafine**

**(C) Olorofim**

**(D) Amphotericin B**

Traditionally, voriconazole (+/−) terbinafine has been the primary antifungal therapy used for the treatment of *L. prolificans* infections (1–3); however, in our patient, even with synergistic testing of voriconazole and terbinafine (susceptibility interpreted as indifferent), this particular strain showed notably elevated MICs across almost all antifungals tested. Based on the susceptibility profile, olorofim appeared to be the most promising antifungal with an MIC of 0.06 μg/mL, so the decision was made to pursue olorofim monotherapy. Of note, in an *in vitro* study reviewing 12 isolates of *L. prolificans*, the MIC range of olorofim was 0.008 μg/mL to >2 μg/mL with a mode of 0.06 μg/mL, which was much lower than the other antifungals tested (voriconazole, itraconazole, isavuconazole, posaconazole, and amphotericin B, all whose modal MIC values were at least 8 μg/mL or above) (4).

The mechanism of action of olorofim (previously known as F901318) is to reversibly inhibit fungal dihydro-orotate dehydrogenase, a crucial enzyme in the pyrimidine biosynthesis pathway (5). This medication is useful in difficult-to-treat, mold infections with high MICs to traditional antifungals, as in our patient. Olorofim’s spectrum of activity includes: *Aspergillus* spp., *Scedosporium* spp., *L. prolificans*, endemic fungi (*Blastomyces*, *Coccidioides*, and *Histoplasma*), *Microascus/Scoulariopsis* spp., *Penicillium*, *Paecilomyces*, *Talaromyces*, and *Madurella* (5); there is variable activity to *Fusarium*, dependent upon particular species (6, 7). Importantly, it should be noted olorofim is ineffective against yeast and *Mucorales* spp. because these organisms have a structurally different version of the target enzyme (5).

**Answer: (C)**

## TREATMENT STRATEGY/PATIENT OUTCOME

Considering no alternative options, discussions with olorofim’s manufacturer, F2G Limited (Manchester, United Kingdom), were initiated, and although this patient did not qualify for their clinical trial due to age, she was accepted for treatment under compassionate use. FDA approval, as well as the University of Utah and Primary Children’s IRB, approved olorofim as an investigational new drug for compassionate use. Based on her body weight of 26 kg, a dosing regimen of 60 mg oral twice daily on day 1, then 30 mg oral twice daily afterward was recommended by F2G’s pharmacokinetic experts. Strict adherence to required labs (liver panel, GGT) was performed as per olorofim’s managed access program protocol provided by Clinigen Direct (US Headquarters: Yardley, Pennsylvania). Additionally, due to limited information regarding pediatric exposure to olorofim, two olorofim trough samples were requested within the first month of therapy, where plasma levels of 0.51 and 0.53 μg/mL were determined; these were above the threshold needed for efficacy (i.e., >0.1 μg/mL) (8).

Due to the logistics in obtaining this medication, olorofim monotherapy was started about 1 month from this patient’s hospital admission and after confirmation of her *L. prolificans* susceptibilities. At that time, her knee continued to remain swollen, and she was having a difficult time walking; however, her CRP was almost normalized and her ESR, although elevated, was downtrending (39 mm/h). Her ESR returned to normal 16 days into her olorofim treatment. Almost 2 months into her olorofim therapy, she participated in a neighborhood kids’ triathlon, and approximately 3 months into her olorofim treatment, her knee swelling had completely resolved.

She had a follow-up knee MRI 4 months into her olorofim therapy, which showed an improved appearance of the patella, with less edema, but full-thickness defect of the overlying articular cartilage.

After review of the literature and in discussion with a fungal expert, the decision was made to treat this patient for a full 6 months with olorofim. Throughout the treatment, she experienced no laboratory or clinical side effects and tolerated the medication well. She had a 1-year follow-up (from initial diagnosis) with Orthopedics, who took x-rays showing progressive healing of the patella. Orthopedics at that time signed off on her care, and she continues to do well with no recurrence approximately 18 months after completion of her olorofim treatment as of publication of this report.

As olorofim is a novel antifungal agent still in its clinical trial phase, limited published data are available regarding its efficacy in treating *L. prolificans* infections; however, as in our case, successes have been reported (9) and the clinical trial data appear promising (10). Furthermore, this case is unique in that pediatric data concerning the use of olorofim and its effects/dosing is even more limited. We hope this paper will help give guidance to other providers treating pediatric patients with *L. prolificans* infections, where olorofim may prove beneficial.
